# Modelling of the Current Density Distributions during Cortical Electric Stimulation for Neuropathic Pain Treatment

**DOI:** 10.1155/2018/1056132

**Published:** 2018-04-23

**Authors:** S. Fiocchi, E. Chiaramello, P. Ravazzani, M. Parazzini

**Affiliations:** CNR Consiglio Nazionale delle Ricerche, Istituto di Elettronica e di Ingegneria dell'Informazione e delle Telecomunicazioni IEIIT, Milan, Italy

## Abstract

In the last two decades, motor cortex stimulation has been recognized as a valuable alternative to pharmacological therapy for the treatment of neuropathic pain. Although this technique started to be used in clinical studies, the debate about the optimal settings that enhance its effectiveness without inducing tissue damage is still open. To this purpose, computational approaches applied to realistic human models aimed to assess the current density distribution within the cortex can be a powerful tool to provide a basic understanding of that technique and could help the design of clinical experimental protocols. This study aims to evaluate, by computational techniques, the current density distributions induced in the brain by a realistic electrode array for cortical stimulation. The simulation outcomes, summarized by specific metrics quantifying the efficacy of the stimulation (i.e., the effective volume and the effective depth of penetration) over two cortical targets, were evaluated by varying the interelectrode distance, the stimulus characteristics (amplitude and frequency), and the anatomical human model. The results suggest that all these parameters somehow affect the current density distributions and have to be therefore taken into account during the planning of effective electrical cortical stimulation strategies. In particular, our calculations show that (1) the most effective interelectrode distance equals 2 cm; (2) increasing voltage amplitudes increases the effective volume; (3) increasing frequencies allow enlarging the effective volume; and (4) the effective depth of penetration is strictly linked to both the anatomy of the subject and the electrode placement.

## 1. Introduction

Neuropathic pain (NP) is defined as pain caused by a lesion or a disease of the central (central neuropathic pain (CNP)) or peripheral (peripheral neuropathic pain (PNP)) somatosensory nervous system [1]. Despite the availability of different drugs, no more than 30–40% patients with chronic NP receive adequate pain reduction (≥50%) by currently available drug-based therapies [2, 3].

Among the nonpharmacological treatments, favourable effects of motor cortex stimulation (MCS) upon CNP have been reported [4–10]. This technique is based on the cortical stimulation with biphasic waves of electric current administered through two (the most used bipolar stimulation) or more electrodes. The stimulating electrode is placed over the motor cortex region where the contralateral painful body area is represented [11–13].

Since its first introduction in 1991 [14] for the treatment of thalamic pain, MCS has been applied worldwide on both animals and humans in the attempt to alleviate medically refractory CNP of different origins, such as the facial NP (including trigeminal neuralgia, trigeminal NP, trigeminal deafferentation pain, symptomatic trigeminal neuralgia, and postherpetic neuralgia), the phantom limb pain, the brachial plexus avulsion, the poststroke pain, the Wallenberg syndrome, the pain secondary to multiple sclerosis, and the posttraumatic brain injury pain (to this purpose see the review studies of both animal and human experiments [15–18]). All these studies have been elaborated based on empirical experience given the incomplete understanding of the pathophysiology of CNP and the difficulties to conduct double-blinded studies; therefore, the mechanism underlying the analgesic effect produced by MCS is still largely debated [11, 19, 20].

However, the most accredited theories addressing pain relief by MCS (for a review see [21]) attribute its efficacy to the neural modulation, given by the flow of current density in the motor cortex [22]. It is indeed believed that the electrical stimulation of the motor cortex inhibits, disrupts, or interferes with the allogenic signals coming from the thalamus and from other hyperactive areas in the brain networks that govern the nociception [14, 23].

In this context, the MCS delivered “dose,” here intended as the current density quantification in the cortex and in the neural tissues, should be considered one of the key points for both the treatment optimization and a deeper understanding of the mechanisms lying behind its efficacy [24].

So far, studies addressing the characterization of the current density distributions due to MCS are scarce and limited to the analysis of different electrode types [25, 26] and/or placed on very simplified cortical models [12, 27–30].

In this study a precise quantification, using computational techniques and appropriately defined metrics, of the current density distributions in the brain of different-aged detailed anatomical human models was conducted. This was performed by reproducing the model of a realistic electrode array for MCS [31] used in clinic and placed on the motor cortex target area that corresponds to the somatic area of pain. Typically, for most of the clinical applications for the neuropathic pain treatment [18], these coincide with facial and upper limb cortical areas, placed in the lower part and in the middle part of the central gyrus, respectively. The electrode array was fed according to the typical frequency and voltage amplitude delivered in the medical practice [7, 9, 10, 32, 33] and the extent to which they affect specific current density-related parameters was quantified. The rigorous analysis of these parameters in realistic and detailed human head models could ultimately help to gain further insights into the evaluation of the clinical outcomes and the optimization of the treatment delivered through the MCS.

## 2. Materials and Methods

### 2.1. Human Models

Three realistic anatomical male models of the Virtual Population Family [34, 35] (Duke, 34 years old; Louis, 14 years old; and Glenn, 84 years old) were used in the study. They were obtained by the segmentation of high-resolution magnetic resonance (MR) images of healthy volunteers and then reconstructed based on a computer-aided design representation of the organ surfaces.

The use of these different anatomical models allowed assessing the characteristics of the current density distributions in three different brain morphologies. In each of them, more than 40 tissues can be distinguished at the head level (head tissues list in Table 1).

**Figure 1:**
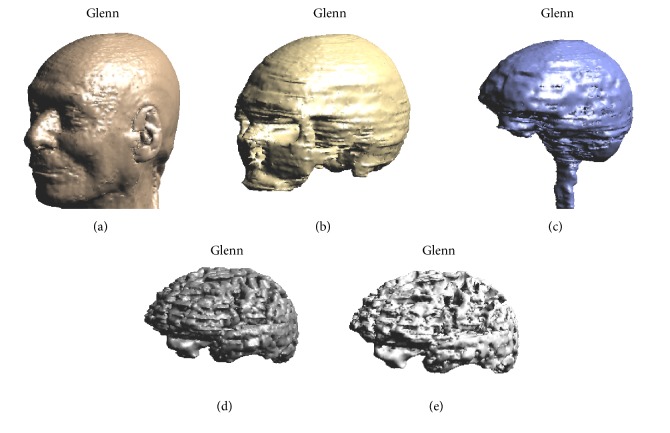
3d rendering of Glenn head model. Tissues shown are (a) skin; (b) skull; (c) CSF; (d) brain grey matter; and (e) brain white matter.

For all the human models, in the regions where the electrodes were positioned, it was possible to distinguish the grey and white brain matter and the CSF (Figure 1). The dielectric properties of each tissue were assigned based on the literature data at low frequency [36, 37]. Table 1 reports the conductivity values of each tissue segmented in the models for each frequency of the stimulation settings used in the study (see Section 2.4 below).

### 2.2. Electrode Modelling

Since the meninges are not segmented in our anatomical models, our computational model is simulating subdural cortical stimulation.

The geometrical and physical characteristics of the electrodes and their position over the cortex have been modelled based on the clinical literature which uses the MCS for the treatment of neuropathic pain (see, e.g., [7, 9, 10, 32, 33]). Briefly, it consists of a four-electrode array of Medtronic, named, Resume II [31]; each electrode has the following properties, as taken from the technical manual: 4 mm diameter and 1.9 mm height disk made of a platinum-iridium alloy (electrical conductivity: 5.278 × 10^6^ S/m). The lower base of each electrode was positioned in contact with the cortex, whereas the side surface and the upper base are covered by a silicone layer (conductivity: 0.1*∗*10^−9^ S/m) mimicking the silicone backing that is not modelled, following the approach already used in literature [25]. A 1 cm distance was kept between two neighbouring electrode centres, according to the manufacturer specifications.

**Figure 2:**
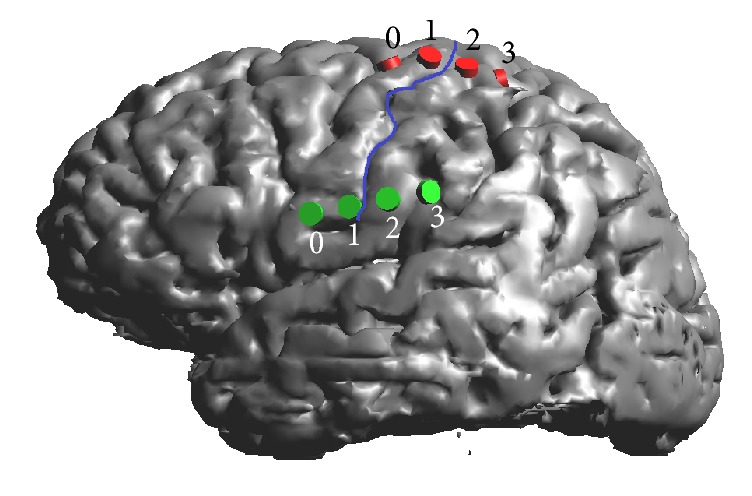
Electrode placement on Duke's cortex over the face target region (green) and over the upper limb target region (red). Blue line shows the central sulcus.

The array was centred on the central sulcus alternatively in two different positions corresponding to the cortical somatotopic representation of the upper limb and of the face. More specifically, the electrodes were placed so that the first and the second (numbered 0 and 1 in Figure 2) lie on the motor cortex over the precentral gyrus, and the third and the fourth (numbered 2 and 3) lie on the somatosensory cortex over the postcentral gyrus and in the proximity of postcentral sulcus, respectively (Figure 2).

### 2.3. Current Density Numerical Simulations

In this study, a simulation-based approach to calculate the current density distributions within brain tissues was performed through the simulation platform SEMCAD X [38], which implements the Finite Element Method (FEM) in the low frequency range. In detail, it solves the Laplace equation to obtain the electric potential (*φ*) distribution:

(1) $$\begin{array}{c} \nabla \cdot \left(\;\sigma \nabla \varphi \;\right)=0, \end{array}$$

where *σ* (S/m) is the electrical conductivity of the human tissues. In the low frequency approximation adopted, ohmic currents dominate displacement currents and capacitive effects are disregarded. The electric field (**E**) and then the current density (**J**) distributions were obtained by means of the following relations:

(2) E=−∇φJ=σE.

Implicitly, we assumed the neuronal excitability in the cortex is proportional to the **J** (and **E**) amplitude and that therefore the regions with a greater current density are more likely stimulated, while the regions with very low current will not directly feel the effects of stimulation [39].

**Figure 3:**
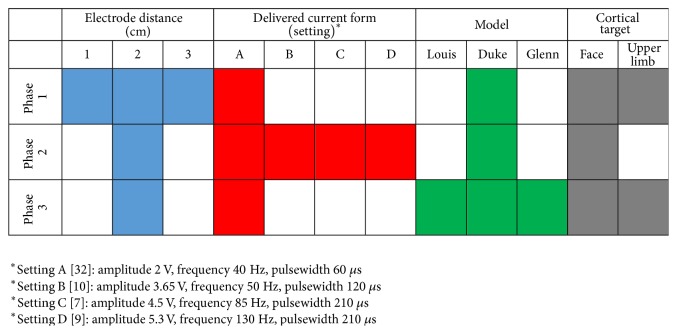
Schematic representation of the simulation scenarios and input signal settings used in the three phases.

For each simulation, the human head model was inserted in a surrounding bounding box filled with air. The tangential *E* field component, *E*_*t*_, was set to be continuous (*E*_*t*1_ = *E*_*t*2_, which is equivalent to *J*_*t*1_/*σ*_1_ = *J*_*t*2_/*σ*_2_) at the interface between two tissues. Current density was set to be parallel to the face at the interface between skin and air. The upper surface of each of the two active electrodes activated was set to a uniform electrical potential and the potential difference between the two electrodes was adjusted according to the settings listed in the following figure (Figure 3). The computation domain was discretized using a uniform rectilinear mesh with a grid discretization equal to 0.5 mm, which assures resolving the thinnest structures of the model. Relative tolerance for FEM convergence was set to 10^−12^. To further optimize the quality of the grid and reduce the calculation time, the computational domain was truncated at the brainstem level. Both these choices (mesh step and dimension of the computational domain) were based on a sensitivity analysis showing that neither reducing the mesh step nor increasing the computational domain had a substantial effect on the field values. At the truncation section, we assigned the boundary condition of continuity of the current, whereas the other faces of the bounding box have been treated as insulated, that is, vanishing flux normal to the computational boundary.

### 2.4. Stimulation Scenarios and Settings

The analysis of the current density distributions was articulated in three distinct phases, corresponding to different scenarios and stimulation settings as summarized in Figure 3.

*Phase 1*. In Phase 1, we evaluated to which extent the interelectrode distance affects the current density distributions on both the target regions. A bipolar electrode configuration was set: the cathode was kept fixed on the electrode positioned on the motor cortical representation of the pain region (i.e., electrode 0 in Figure 2), whereas the anode was changed in each simulation from electrodes 1 to 3 of the four-electrodes array (see Figure 2) so that the distance between the two active electrodes varied from 1 cm to 2 cm and 3 cm. This was done for both motor cortex targets, placing the electrode array alternatively on these two regions (i.e., once on the face and once on the upper limb cortical somatotopic representation). The potential difference between the active electrodes was kept fixed at 2 V and the stimulation frequency equal to 40 Hz (in the following these setting parameters will be referred to as “Reference”), as commonly used in chronic MCS therapy for neuropathic pain [5, 32] and the simulation was performed on the 34-year-old model (“Duke”).

*Phase 2*. In Phase 2, we analysed the effect of both the amplitude and the frequency stimulus for a fixed interelectrode distance of 2 cm. In particular, the potential difference between cathode (Electrode 0 placed over the precentral gyrus in the motor cortex, Figure 2) and anode (Electrode 2 on postcentral gyrus in the somatosensory cortex, Figure 2) and the frequency of the stimulation signal have been varied in the range described by following literature studies:

- Setting A: taken from [32], who found a significant improvement in the clinical assessment for the evaluation of NP of various origins when the stimulation settings were as follows: amplitude, 2 V; frequency: 40 Hz; and pulsewidth, 60 *μ*s.
- Setting B: based on [10], who established the efficacy of electric stimulation on a central poststroke facial pain subject, with the following stimulation parameters: amplitude, 3.65 V; frequency, 50 Hz; and pulsewidth, 120 *μ*s.
- Setting C: [7] reported the successful application of bipolar MCS (amplitude: 4.5 V, frequency: 85 Hz, and pulsewidth: 210 *μ*s) to patients suffering from thalamic neuropathic pain (TNP) and poststroke pain (PSP).
- Setting D: [9] obtained a positive pain relief in 27 patients affected by chronic neuropathic pain, by increasing both amplitude and frequency and pulsewidth of the delivered pulse up to 5.3 V and 130 Hz and 210 *μ*s, respectively.

Simulations were run by varying, for each stimulus amplitude (i.e., 2 V, 3.5 V, 4.5 V, and 5.3 V), all the four stimulus frequencies used in the above-mentioned clinical studies (40, 50, 85, and 130 Hz), thus allowing us to consider all the possible combinations of the two parameters. The stimulation signal was considered as a pure sinusoid, following an approach already used in the literature [25, 26]. The pulsewidth was then considered only to verify the stimulation stayed within the safety margins to avoid neuronal damage (see in Section 4 below). Also in this phase, the electrode array was placed over the face motor cortex area of the 34-year-old model (Duke).

*Phase 3*. The third phase was designed to investigate the age-dependent anatomical differences of the current density distributions generated in the cortex. Bipolar stimulation at the intensity equals 2 V and frequency to 40 Hz was delivered at the electrodes 0 and 2 (interelectrode distance of 2 cm) placed on Duke (34 years old), Louis (14 years old), Glenn (84 years old) on both cortical targets of the face and of the upper limb.

### 2.5. Data Analysis

The current density distributions were assessed in the cortex and in the white matter. The following parameters were then calculated, according to the study performed by Kim's group ([25–27, 40, 41]):

- Effective volume (EV_50_): evaluated for both grey and white matter, it is the volume which has a current density greater than 50% of motor cortex threshold (MCT) (*J*_MCT_ = 2.5 A/m^2^ as calculated by [42]). That threshold was chosen as the current density amplitude that can provoke analgesia without motor effects and lies in the range of the practical usage case [27]. This index therefore quantifies the tissue volume that undergoes neural modulation.
- Effective depth of penetration (*D*_*J*MCT_): it is the maximum depth [mm] from the cerebral cortex surface, reached by a current density higher than the 50% of *J*_MCT_.

These indexes were analysed as surrogate of the stimulation effectiveness.

## 3. Results

**Figure 4:**
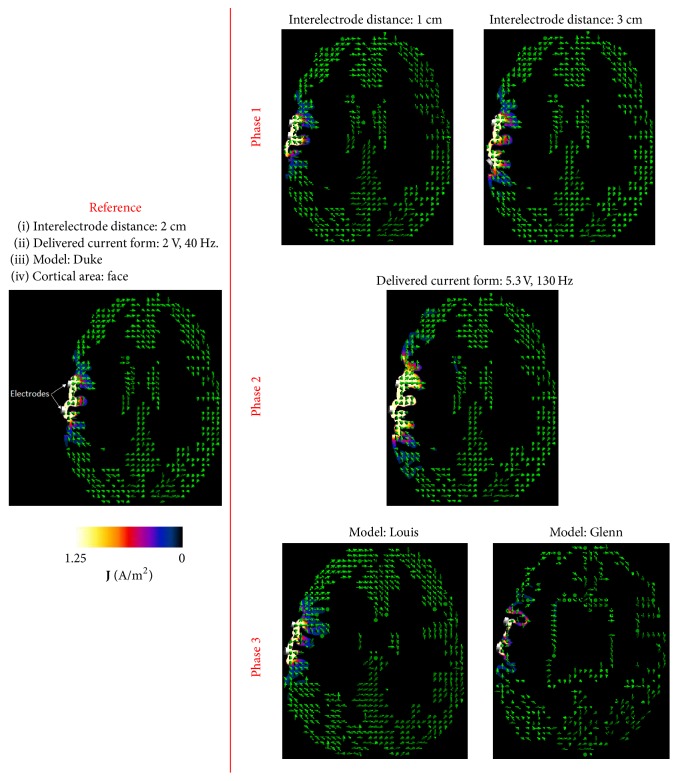
Examples of current density (*J*) distributions over the axial slices at 4 cm below the projection of Cz over the cortex. Colormaps are set between 0 and the 50% of MCT (i.e., 1.25 A/m^2^); therefore, white areas correspond to the areas in which *J* is equal or higher to the scale upper limit. Green arrows represent the direction of the current density on the cortex. Panel on the left shows the *J* distribution produced in the cortex by “Reference” simulation.

Figure 4 shows some examples of the current density distributions over the axial slice at 4 cm below the projection of Cz over the cortex (where Cz is referred to according to the 10–20 EEG system) for some phases of the different stimulation settings. The colour maps represent the amplitude distribution of **J** and are all clipped above 50% of the *J*_MCT_ to favour the comparison between the amplitude distributions resulting from the settings used in the three different phases. The green arrows represent the direction of **J**, which is preferentially directed tangentially in the crowns of the gyri, whereas it is directed predominantly normally at the bottom of the sulci. In the following, the results will be presented by investigating the two indexes described above, and within each index analysis, by comparing the results of the three phases.

### 3.1. Effective Volume (EV_50_)

**Figure 5:**
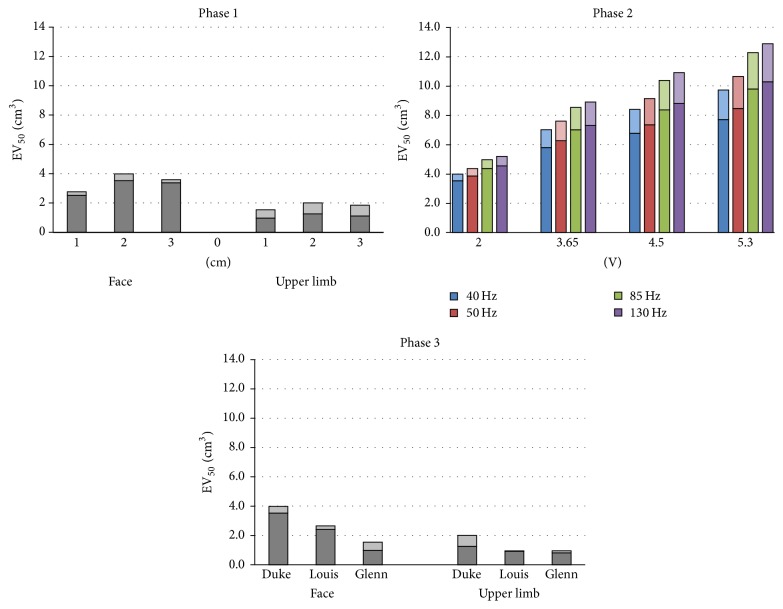
Effective volume calculated from the current density distributions by varying the interelectrode distance and positioning (Phase 1), delivered signal settings (Phase 2), and models and positioning (Phase 3). Each bar is the sum of the cortex effective volume (dark) and the white matter effective volume (light).

Figure 5 shows the effective volume (in cm^3^) calculated varying the stimulation settings according to the three phases (see Section 2.4 above). The bars in the graphs are the sum of the cortex effective volume (dark colours) and the white matter effective volume (light colours). The total effective volume (EV_50_) (i.e., the sum of cortex and white matter effective volume) for each simulation setting can be therefore read on the vertical axis. From that figure, one can notice that for a 2 cm interelectrode distance (Phase 1), the total effective volume is significantly higher (up to 30%) than the same quantity calculated for the other two distances and for both the cortical areas. Moreover, one could identify a comparable trend of the EV_50_ along the three distances for both the cortical regions. Similar considerations apply when we consider separately the effective volume trends on the cortex and white matter. Interestingly, the total effective volumes calculated on the facial cortical area are almost double the ones calculated on the upper limb area. This is mainly due to the contribution of the cortex effective volume, whereas the white matter effective volume is higher in the upper limb area. However, for both the electrode placements and for all the distances, the total effective volume stays below 4 cm^3^. This result changes when increasing amplitude and frequency stimulus (Phase 2). In particular, increasing amplitude, the ratio between EV_50_ calculated with an applied potential difference of 3.65 V, 4.5 V, and 5.3 V with respect to EV_50_ calculated at 2 V equals 1.73, 2.10, and 2.46, respectively, independently of the stimulus frequency. Similarly, increasing frequency, the trend of the EV_50_ is the same for each applied potential difference. The ratio between EV_50_ calculated at 50, 85, and 130 Hz with respect to EV_50_ calculated at 40 Hz equals 1.1, 1.25, and 1.3, independently of the applied potential difference and that happens even for both cortex and white matter proportions.

On the contrary, the effective volume variation does not present a clear age-related trend across the three models (Phase 3). The levels, however, for both the targets, decrease in the younger (i.e., Louis) and older (i.e., Glenn) male model with respect to the adult male (i.e., Duke), and hence in this phase the total EV_50_ stays everywhere below 4 cm^3^.

### 3.2. Effective Depth of Penetration (*D*_*J*MCT_)

**Figure 6:**
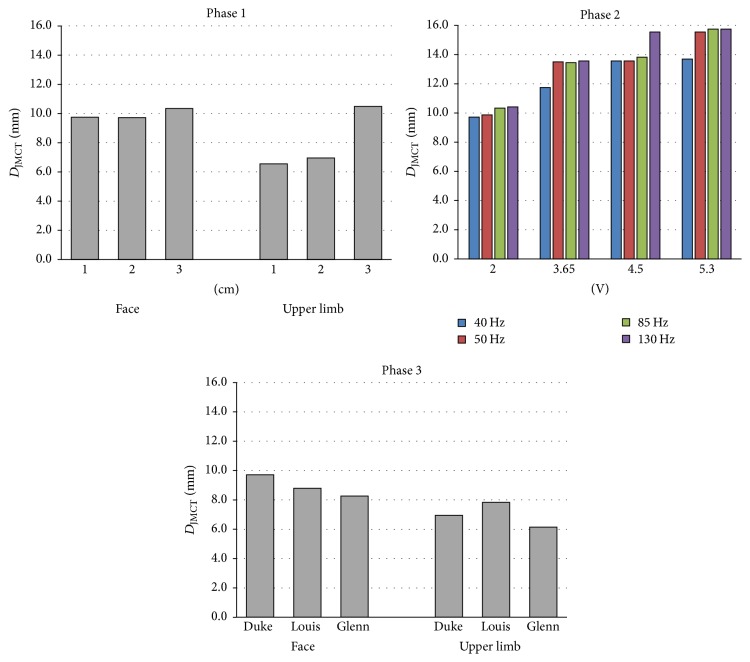
Effective depth of penetration calculated from the current density distributions by varying the interelectrode distance and positioning (Phase 1), the delivered signal settings (Phase 2), and the models and positioning (Phase 3).

Figure 6 shows the penetration depth (*D*_*J*MCT_ in mm) calculated varying stimulation scenarios and settings of the three phases. This analysis was conducted considering the whole brain matter, that is, the cortex and the white matter taken together. By increasing the interelectrode distance (Phase 1), the penetration depth increases with some differences between the two cortical areas: in the face cortical area, the capability to penetrate the cortex ranges from 9.7 to 10.3 mm, whereas in the upper limb from 6.5 to 10.5 mm.

The increase in both amplitude and frequency (Phase 2) results in penetration depth increase, but no clear trend of those increases can be identified. Moreover, *D*_*J*MCT_ calculated on the old male model (Glenn) is reduced in both the cortical regions of about 15–20% with respect to the 34-years-old male model (Phase 3).

## 4. Discussion and Conclusions

### 4.1. Motor Threshold Definition

Although MCS for the neuropathic pain treatment is under investigation for more than two decades, the mechanism of action behind its effectiveness is still not clear. Moreover, no guidelines for the best set of stimulation parameters and electrode montages exist so that all adjustments to them for improving MCS results still depend on iterative empirical testing [24, 43] and are highly individualized.

Lately, different authors [15, 24, 43] have proposed that individual stimulation parameters can be predicted as a percentage of motor threshold activation. A simple way to assess the reliability of this approach can be provided by numerical simulation, actually consistent with experimental data [26].

As discussed in the clinical neurobiology literature [44, 45] the mechanism underlying both the damaging and nondamaging effects of the stimulation are associated with the synchronous activity of a substantially large number of neurons. That supports that the modulation effects are driven by some “mass action” gathered from the simultaneous activation of a critical neuronal volume. Although in our simulations the maximum levels of current density exceed the MCT (see, i.e., white areas in Figure 4), the cortical volume with a current density higher than the MCT is very small and limited to a maximum volume of 1.5 cm^3^ (across all the stimulation settings and scenarios examined) distributed mainly under the cathode and the anode. This volume, given that MCS clinical studies that had used the same stimulation parameters did not report side effects [7, 9, 10, 32, 33], is unlikely to induce a neural “mass action” which would lead to an undesired motor response. According to these considerations, in our study we used the 50% of the MCT as a modulation threshold and hence the level that limits the analgesic effect and the efficacy of the technique. However, the goodness of this threshold is an important key factor to be validated in further studies, also in view of the highly individualized actual device settings and the fact that here we modelled the subdural stimulation instead of the epidural stimulation from which stimulus parameters (mainly amplitude and frequency) are taken. One should also note that the MCS threshold was estimated by a transcranial magnetic stimulation (TMS) based study [42]: therefore, given the different *J* spatial derivative in TMS compared to MCS, it cannot be excluded that neuronal activation in subdural cortical stimulation could be also predicted by different metrics, such as its spatial derivative, as done in previous studies based on the activating function [12, 28, 30, 46]. The authors of [42] numerically calculated the minimum current density peak in the cortex that elicits motor activation from which they estimated the rheobase current and the chronaxie time to determine the strength-duration curve. In the frequency range considered in this study, it slightly varies between 2.49 A/m^2^ and 2.62 A/m^2^. This range is in line with the levels for motor cortical activation proposed in the electric stimulation literature, and the 20–50% of that quantity (i.e., 0.5–1.5 A/m^2^) corresponds to the widely accepted range for motor cortex modulation administered through direct current stimulation [30, 47–53].

### 4.2. Effective Volume Analysis

The first index of efficacy analysed, that is, the effective volume, allows quantifying the percentage of volume of the cerebral cortex and of the white matter that responds to the stimulus. In other words, higher volumes correspond to a broader area stimulated. From Figures 4 and 5, one can notice that, among the three interelectrode distances tested, in both the target areas and particularly for the facial cortical region, the 2 cm interelectrode distance can stimulate a broader volume. This is probably due to the anatomy of the cerebral cortex that, with its typical convolutions, strongly influences the current density flow, preventing a trivial prediction of its distribution. In an isotropic spherical model, an increasing interelectrode distance linearly raises the values of the volumetric parameters. Conversely, using a realistic anatomical model we show that it happens only as long as the second electrode does not fall in a sulcus of the motor cortex. The central sulcus, which separates the cathode and the anode, prevents indeed that the totality of the area of the cortex between the two electrodes feels a current density sufficient to generate motor cortex neural excitation.

Similarly, the anatomical differences between the different-aged models considerably change both the pattern of stimulation and the indexes examined, with a net contraction of the effective volume in the older men (Figures 5 and 6). That is probably due to the cortical atrophy that usually affects the elderly [51, 52], producing an enlargement of the subarachnoid space (average CSF thickness at the electrodes levels is 0.21 mm, 0.94 mm, and 3.46 mm for Louis, Duke, and Glenn, resp.) and an increase of the CSF volume filling the empty space in the sulci (total CSF volume increases from 214 to 333 and to 619 cm^3^ in Louis, Duke, and Glenn, resp.). Increasing CSF volume/thickness has a strong impact in epidural cortical stimulation by reducing the amount of current penetrating the cortex and hence reducing the stimulation efficacy, as reported in previous studies [12, 28, 29], but has little effect on the total current entering grey matter in subdural cortical stimulation [41]. However, it is important to consider the CSF volume-related shunting effect when translating the present study's results to epidural motor cortex stimulation.

The thinning of the cortex in Glenn (up to 1.5 mm cortical thickness decrease), visible also in Figure 4, enlarges the space between the convolutions (the sulci) and reduces the spread of the current density in the cerebral cortex in the direction perpendicular to the sulcus, thus increasing the focality of stimulation and hence reducing the effective volume. This is in line with previous modelling studies showing that anatomical differences would highly affect the stimulation efficacy in epidural cortex stimulation [12, 28] and further enforce the need to use anatomical and detailed human models in computational studies addressing current density distribution quantification.

In the case in which it is necessary to stimulate a larger area, our results suggest that the frequency or the amplitude of the stimulus should be increased (Figure 5, Phase 2). The increasing rate of the effective volume scales with the increasing rate of the stimulus amplitude and it agrees with previous calculations performed in two similar studies by Kim's group [27, 41], whereas it is much slower with respect to the increasing rate of the stimulus frequency. Limited effects of frequency could be given by the conductivity variation (Table 1), which however weakly contributes to the electric field distribution variability [54]. However, one should also take into account the fact that increasing frequencies could have other effects on neural activation such as the ones linked to the selective fibers recruitment or to the indirect/synaptic activation of neurons [55].

### 4.3. Effective Penetration Depth Analysis

The effective volume is strictly linked with the second index examined: the effective penetration depth (Figure 6). As with the previous index, it is greatest in the facial area (Phase 1 and Phase 3). It increases with increasing interelectrode distance, in particular in the upper limb cortical area, when the anode falls into the postcentral gyrus. However, one can notice its limited variability across the different interelectrode distances and across the models. Indeed, this index, which is a sulci-parallel propagation index, is less affected by the elderly cortical atrophy compared to the previous index.

As to the stimulus settings (Figure 6, Phase 2), the effective penetration depth increases very slowly with the frequency increase. While the increase in the frequency can more effectively enlarge the stimulation (Figure 5, Phase 2), it cannot deepen it. The only way to improve that distance of about the 50% within the settings range examined here is to increase the amplitude of the stimulus.

### 4.4. Relationship between Stimulation Efficacy Related Indexes and Simulation Scenarios/Settings

Table 2 summarizes the main relationships between the indexes and the stimulation scenario and settings analysed.

In terms of absolute values, the indexes of efficacy here evaluated are in good agreement with the values calculated by Kim and colleagues [41] with similar electrode geometry (4 mm versus 5 mm diameter), montage (interelectrode distance of 1 cm, placement over the upper limb area), and stimulation parameters (delivered voltage 2 V at 50 Hz). However, one should take into account the fact that the impact of using typical epidural cortical stimulus settings for a subdural cortical stimulation could not be negligible in terms of both current density values and spatial distribution. As a result of the first remark, the effective volume, the depth of penetration, and the cortical volume above MCT are most likely overestimated here compared to epidural cortical stimulation, thus representing a conservative estimate of side effects related to motor activation in case of epidural cortical stimulation. In addition, moving the electrode from above the dura to directly on the cortex strongly affects also the current density spatial derivative, which in turn impacts on activation of axons (i.e., activating function).

### 4.5. Stimulation Settings Selection and Safety Related Issues

In the clinical practice, the stimulation settings are chosen based on the available literature and are often modified during the treatment according to the individual response [43]. The need to use, for some patients, increasing frequency and amplitude is indeed probably driven both by the ineffectiveness of the stimulation when the starting settings are used and by the different patients' motor thresholds. As discussed above, the motor threshold was here kept fixed but the intersubjects physiological variability here examined has to be taken into account when we move from modelling to practice. In the same way, the upper limit of the stimulation settings should assure a sufficient risk margin to prevent the tissue damage. That is indeed the main constraining factor in the choice of stimulation settings for implantable devices used for the treatment of neurological disorders [56]. However, here we verified that the stimulation parameters used in our study stays within the recommended safety margins. Specifically, we evaluated the charge per phase (A·s) and the charge density per phase (measured on the surface of the electrode) (A·s/cm^2^) for each combination of amplitude and pulsewidth tested and we compared them with the couple limit values reported in the literature [56, 57]. It is believed indeed that both are factors that synergistically determine the stimulation threshold that induces neuronal damage [57]. The results of this analysis (not shown here in detail), indicated, however, that all the possible setting combinations here modelled produce a couple of charge per phase and charge density per phase that guarantees the compliance with the safety limits.

This discussion therefore confirms that our computational study can give important indications about the spatial distribution of the current density during MCS. The use of detailed anatomical models provides indeed a substantial advance in the computational results reliability and have the potential to be included in forward models [58], similar to that used to solve inverse problems in electroencephalography (EEG) analysis, for providing important suggestions about the planning of a more focused stimulation strategy.

## Figures and Tables

**Table 1:** Conductivities (S/m) of the head tissues at different stimulation frequencies [36, 37].

Tissue	Conductivity (S/m)			
	40 Hz	50 Hz	85 Hz	130 Hz
Air internal	0	0	0	0
Artery, blood vessels, vein	0.7	0.7	0.7	0.7
Mandible, marrow red, skull, teeth, vertebrae	0.0201	0.0201	0.0201	0.0201
Brain grey matter, hippocampus, hypothalamus, thalamus	0.0681	0.0753	0.0869	0.0915
Brain white matter, commissure	0.0506	0.0533	0.0573	0.0590
Cartilage, ear cartilage, intervertebral disks,	0.171	0.171	0.172	0.172
cerebellum	0.0881	0.0953	0.107	0.111
Cerebrospinal Fluid (CSF)	2	2	2	2
Connective tissue, tendon, ligament	0.263	0.270	0.295	0.322
Cornea,	0.421	0.421	0.422	0.422
Muscle	0.224	0.233	0.259	0.278
Ear skin, skin	0.0002	0.0002	0.0002	0.0002
Eye lens,	0.321	0.321	0.322	0.323
Eye sclera	0.503	0.503	0.503	0.503
Eye vitreous humor	1.5	1.5	1.5	1.5
Fat, subcutaneous adipose tissue (SAT)	0.0188	0.0196	0.0206	0.0210
Hypophysis, pineal body	0.521	0.521	0.522	0.523
Medulla oblongata, midbrain, pons	0.0594	0.0643	0.0721	0.0753
Mucosa	0.00042	0.00043	0.00045	0.00048
Nerve, spinal cord	0.0269	0.0274	0.0280	0.0281
Tongue	0.271	0.271	0.272	0.272

**Table 2:** Schematic representation of the main findings of this study.

	EV_50_	*D*_*J*MCT_
	(Figure 5)	(Figure 6)
Interelectrode distance (Phase 1)	Max @ 2 cm	↑ with distance
Stimulus amplitude (Phase 2)	↑↑ with amplitude	↑ with amplitude
Stimulus frequency (Phase 2)	↑ with frequency	↑ with frequency
Age (Phase 3)	No clear trend with age (less in the elderly)	No clear trend with age (but min in the elderly)

Neuronal response was activated in the volume where **J > 50% MCT** (→ modulation threshold); ↑EV_50_→  ↑ spread of modulated tissue; ↑*D*_*J*MCT_→  ↑  depth of modulated tissue.
